# M6A methylation of DEGS2, a key ceramide-synthesizing enzyme, is involved in colorectal cancer progression through ceramide synthesis

**DOI:** 10.1038/s41388-021-01987-z

**Published:** 2021-08-06

**Authors:** Wei Guo, Cuiyu Zhang, Panpan Feng, Mingying Li, Xia Wang, Yuan Xia, Dawei Chen, Jingxin Li

**Affiliations:** 1 Department of Colorectal Surgery, Qilu Hospital, Cheeloo College of Medicine, Shandong University, Jinan, Shandong China; 2 Department of Physiology, School of Basic Medical Sciences, Cheeloo College of Medicine, Shandong University, Jinan, Shandong China; 3 Department of Hematology, Qilu Hospital, Cheeloo College of Medicine, Shandong University, Jinan, Shandong China; 4 Laboratory of Medical Chemistry, Interdisciplinary Cluster for Applied Genoproteomics (GIGA) Stem Cells, Faculty of Medicine, University of Liège, CHU, Sart-Tilman, Liège, Belgium

**Keywords:** Colorectal cancer, Predictive markers

## Abstract

N6-methyladenosine (m6A) is the most prevalent RNA epigenetic regulator in cancer. However, the understanding of m6A modification on lipid metabolism regulation in colorectal cancer (CRC) is very limited. Here, we observed that human CRCs exhibited increased m6A mRNA methylation mediated by dysregulation of m6A erasers and readers. By performing methylated RNA-immunoprecipitation sequencing (MeRIP-seq) and transcriptomic sequencing (RNA-seq), we identified DEGS2 as a downstream target of m6A dysregulation. Overexpression or knockdown of DEGS2 confirmed the role of DEGS2 in proliferation, invasion and metastasis of CRC both in vitro and in vivo. Mechanistic studies identified the specific m6A modification site within DEGS2 mRNA, and mutation of this target site was found to drastically enhance the proliferative and invasive ability of CRC cells in vitro and promote tumorigenicity in vivo. Lipidome analysis showed that lipid metabolism was dysregulated in CRC. Moreover, ceramide synthesis was suppressed due to DEGS2 upregulation mediated by m6A modification in CRC tissues. Our findings highlight that the function of DEGS2 m6A methylation in CRC and extend the understanding of the importance of RNA epigenetics in cancer biology.

## Introduction

Colorectal cancer (CRC), also known as large bowel cancer, is one of the most prevalent malignancies in adults; it ranks third in the incidence rate and ranks fifth among the leading causes of tumor-related death worldwide [1]. Epidemiological data have revealed that the 5-year survival rate of CRC patients ranges from 90% for patients with stage I disease to 10% for those with metastatic disease [2]. Owing to the high rate of recurrence and metastasis, CRC patients commonly have a poor prognosis, especially in advanced stages. Although treatments for CRC, ranging from complete mesocolic excision, neoadjuvant chemoradiotherapy to targeted therapy or immunotherapy, have immensely progressed over recent decades, the outcomes of CRC are still unsatisfactory. Thus, it is imperative to further elucidate the molecular pathogenesis of CRC to develop novel therapeutic strategies and reduce the mortality rate of this malignancy.

N6-methyladenosine (m6A) methylation has been identified as the most abundant modification ubiquitously occurring in eukaryotic mRNAs [3]. The effects of m6A modification include the regulation of mRNA stability, splicing, transport, localization, translation, and the regulation of RNA–protein interactions [4]. m6A modification is dynamic and reversible in mammalian cells, and it can be installed by m6A methyltransferases (also called writers, including METTL3, METTL14, and WTAP) and removed by m6A demethylases (also called erasers, including FTO and ALKBH5). In addition, specific RNA-binding proteins (also called readers, including YTHDF1/2/3, eIF3, IGF2BP1/2/3, and HNRNPA2B1) can bind to the m6A motif directly or indirectly to affect RNA function. In the past few years, the biological functions of m6A modulators have been reported to be associated with stem cell differentiation, tissue development, circadian periods, and tumor progression [5].

m6A modification is implicated in diverse biological processes, especially tumorigenesis. Several members (e.g., METTL3, METTL14, FTO, ALKBH5, and YTHDF2) actively participate in human cancers such as acute myeloid leukemia [6], glioblastoma [7], breast cancer [8], and endometrial cancer [9]. Multiple functions, ranging from tumor initiation, development, and metastasis to cancer stem cell pluripotency, are mediated by m6A methylation. METTL3 is reported to act as an oncogene and to maintain SOX2 expression through an m6A-IGF2BP2-dependent mechanism in CRC cells, indicating a potential biomarker panel for prognostic prediction in CRC [10]. In addition, METTL14 is primarily reported to act as a tumor suppressor by manipulating the processing of the m6A-modified lncRNA XIST with the aid of the m6A reader protein YTHDF2 [11]. Nevertheless, the role of m6A methylation in metabolism, especially lipid metabolism, is poorly understood in CRC.

Sphingolipids are structural molecules of cell membranes with important roles in maintaining barrier function and fluidity [12]. Sphingolipids also regulate various biological processes such as growth, proliferation, migration, invasion and/or metastasis by controlling signaling functions within the cancer cell signal transduction network [13]. For example, the generation of ceramide (Cer) and sphingosine is induced by chemotherapy, radiation, and/or oxidative stress, and these sphingolipids mediate cell death, senescence, and/or cell cycle arrest [14].

De novo biosynthesis occurs in the endoplasmic reticulum and involves a highly conserved sequence of enzymatic reactions. Delta 4-desaturase sphingolipid 1 (DEGS1) is the key enzyme that oxidizes dihydroceramide (dhCer) to Cer, which is the last step of this biosynthetic pathway. Identified as a DEGS1 homolog, DEGS2 is the major enzyme that catalyzes the conversion of dhCers to phytoceramides [15]. An increasing number of studies have linked Cer metabolism to various biological processes, especially cancer development, indicating the value of evaluating the potential of therapeutic strategies targeting the enzymes that catabolize Cer.

In the present study, the Cer-synthesizing enzyme DEGS2, which displayed markedly increased expression levels in CRC tissues compared to normal colorectal tissues, was identified and selected for further validation and functional analysis in the context of CRC progression. We revealed the biological effect of m6A modification on DEGS2 and Cer metabolism in CRC. We proposed that m6A modification of DEGS2 correlated with CRC progression and maybe a novel predictive biomarker and therapeutic target for CRC.

## Results

### CRC is associated with high levels of m6A mRNA methylation

**Fig. 1:**
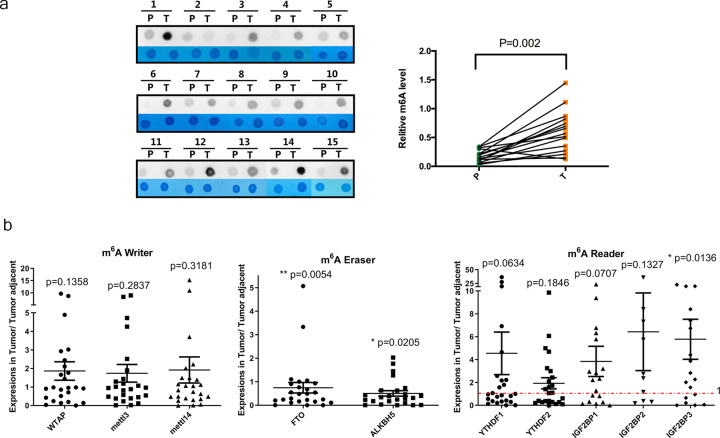
CRC is associated with high levels of m6A mRNA methylation. **a** The mRNAs isolated from CRC tissues and paired normal colorectal mucosa samples were assessed by dot blot analyses with an anti-m6A antibody, and methylene blue (MB) stain served as the loading control (representative images in left panel). The relative m6A content on mRNA in CRC tissues and paired normal colorectal mucosa samples were calculated (right panel, *n* = 15). **b** Scatter plot of the expression levels of WTAP, METTL3, METTL14, FTO, ALKBH5, YTHDF1, YTHDF2, IGF2BP1, IGF2BP2, and IGF2BP3 in tumor tissues relative to tumor-adjacent tissues; *n* = 25 tumor-normal tissue pairs for the assessment of the m6A writers, erasers, and readers listed above. The *p* values were determined by two-tailed *t*-test.

To elucidate the functional roles of m6A modification in CRC, we first examined the m6A RNA levels in 15 CRC tissues and paired normal colorectal mucosa samples. We found that the m6A RNA levels were significantly higher in CRC tissues via a dot blot assay (Fig. 1a). m6A modification is mainly catalyzed by m6A writers, erasers, and readers. We hypothesized that the increased m6A RNA levels in CRC were caused by the dysregulation of writers, erasers, and readers. We then compared the mRNA levels of key m6A writers (METTL3, METTL14, and WTAP), erasers (ALKBH5 and FTO), and readers (YTHDF1, YTHDF2, IGF2BP1, IGF2BP2, and IGF2BP3) in 25 pairs of CRC and paired normal colorectal tissues by RT-qPCR (Fig. 1b). Primer sequences for qPCR analysis were listed in Table S1. We found that a majority of CRCs exhibited significantly reduced expression of the m6A demethylases FTO and ALKBH5, and increased expression of the m6A reader IGF2BP3 compared to that in adjacent normal tissues. Taken together, these results suggest that a large portion of human CRCs exhibit increased m6A mRNA methylation mediated by dysregulation of m6A erasers and readers.

### Variations in m6A-regulated genes in CRC

**Fig. 2:**
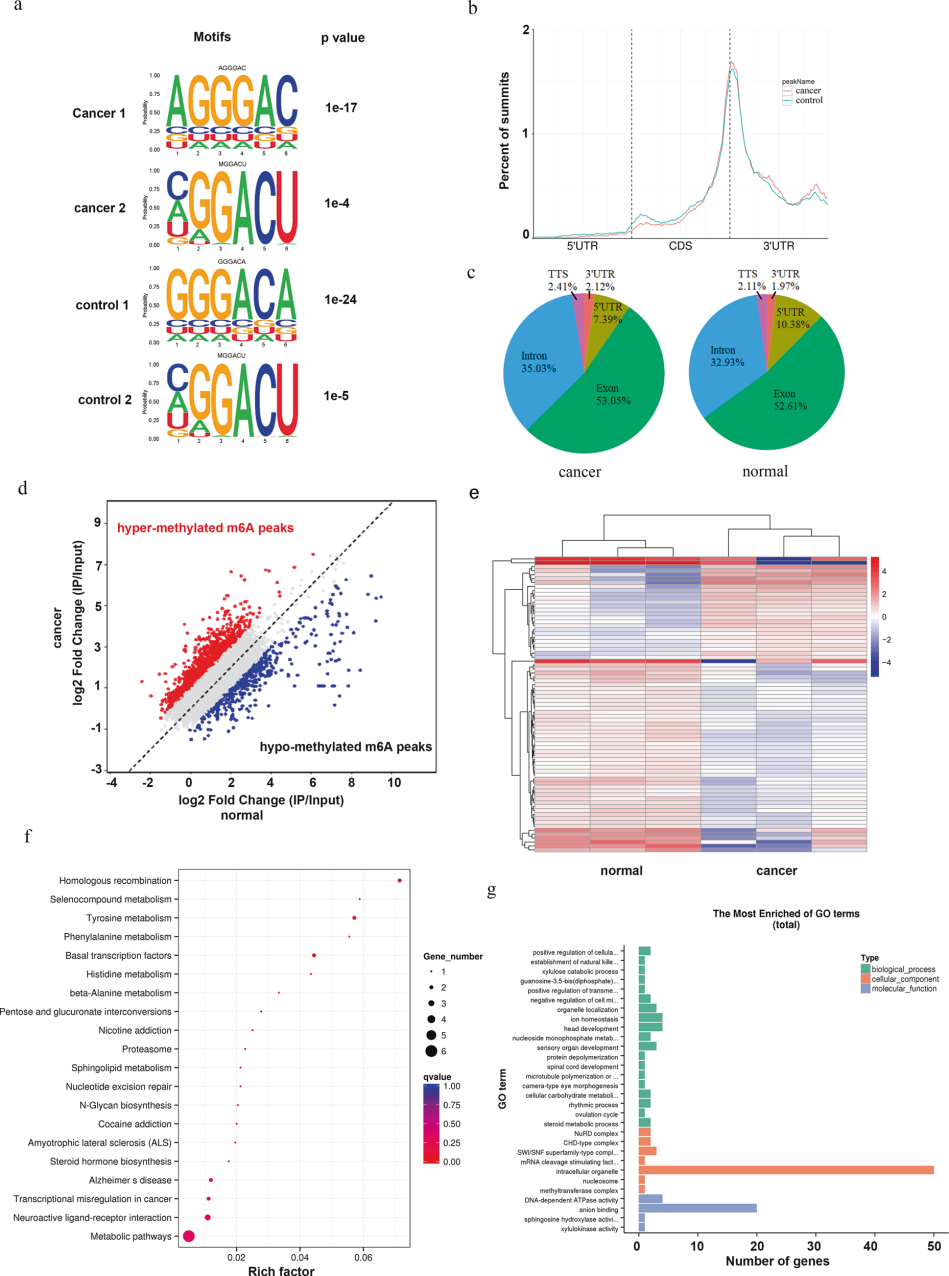
Variations in m6A-regulated genes in CRC. **a** The predominant consensus motif GGAC was detected in both the control and CRC samples by m6A-seq. **b** Density distribution of m6A peaks across mRNA transcripts. Portions of the 5′ untranslated region (5′ UTR), coding sequence (CDS), and 3′untranslated region (3′ UTR) were split into 100 segments, and then the percentages of m6A peaks that fell within each segment were determined. **c** m6A peak distribution in the 5′ UTR, start codon, CDS, stop codon or 3′ UTR across the entire set of mRNA transcripts. **d** Scatter plot for hypermethylated m6A peaks and hypomethylated m6A peaks in CRC tissues compared with control tissues. ‘Hypermethylated m6A peaks’, m6A peaks which are higher in CRC tissues compared to control tissues. ‘Hypomethylated m6A peaks’, m6A peaks which are lower in CRC tissues compared to control tissues. **e** Heatmap of 75 genes, which shows 1.5-fold m6A-mediated expression alteration in CRC tissues compared with control tissues. **f**, **g** The cluster Profiler packages identified the enriched GO and KEGG processes of 75 genes, which showed a 1.5-fold m6A-mediated expression alteration in CRC compared with the control.

To investigate the variations in the m6A modification of specific genes, we mapped the m6A methylomes of CRC tissues and paired normal colorectal mucosa samples by m6A sequencing (m6A-seq) with independent biological replicates. m6A occurs mostly in the RRACH (R=G or A, H=A, C or U) consensus sequence. Our MeRIP-seq results revealed that the GGACU motif was highly enriched within m6A sites in both CRC tissues and paired normal colorectal mucosa samples (Fig. 2a). m6A peaks were especially abundant in the vicinity of stop codons (Fig. 2b). m6A-seq analysis identified 11,951 and 13,031 m6A peaks from CRC tissues and paired normal colorectal mucosa, respectively. We further analyzed the total m6A distribution patterns of mRNAs according to the m6A-seq results. Similar patterns were observed in the total m6A distribution in CRC tissues and paired normal colorectal mucosa samples (Fig. 2c), showing that m6A peaks were mainly enriched in exon regions. A scatter plot showed abundant hypermethylated and hypomethylated m6A peaks in both CRC tissues and paired normal colorectal mucosa samples (Fig. 2d).

We compared the CRC tissues to paired normal colorectal mucosa samples and identified a total of 75 genes with a 1.5-fold m6A change in the expression level and *p* value less than 0.05 (Fig. 2e). Kyoto Encyclopedia of Genes and Genomes (KEGG) enrichment analysis of these 75 genes indicated that several genes were associated with metabolic pathways as well as amino acid metabolism and sphingolipid metabolism (Fig. 2f). Gene ontology (GO) enrichment analysis showed that a great proportion of the differentially m6A-methylated genes was associated with intracellular organelles (Fig. 2g). Collectively, the m6A-seq data revealed that m6A modifications occur on several genes related to amino acid metabolism, sphingolipid metabolism, and other metabolic pathways during colorectal carcinogenesis.

### DEGS2 is involved in m6A-regulated carcinogenesis in CRC

**Fig. 3:**
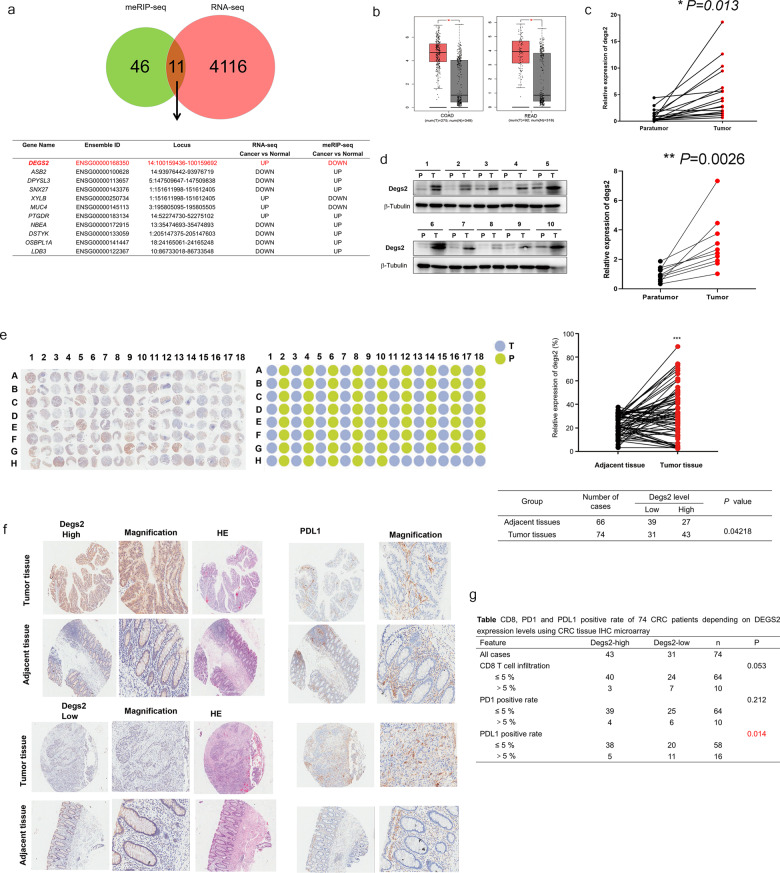
DEGS2 is involved in m6A-regulated carcinogenesis in CRC. **a** Overlapping genes with twofold expression changes in the RNA-seq and m6A-seq analyses. **b** The relative expression of DEGS2 in colon (left) and rectal (right) cancer tissues and their corresponding adjacent normal tissues based on data available from the TCGA database. **c**, **d** Expression of DEGS2 in paired human CRC tissues and adjacent normal mucosa tissues (*n* = 17) by RT-qPCR and WB. **e** Images of IHC staining of DEGS2 in 86 CRC tissues and 78 adjacent normal tissues and quantification analysis of DEGS2 in CRC tissues compared with the control tissues. **f** Representative images of IHC staining of DEGS2 and PDL1 and hematoxylin-eosin (HE) staining. The high (upper) and low (lower) expression levels of DEGS2 were evaluated semiquantitatively by recording the staining intensity (high score: 27–90; low score: 0–27). **g** Chi-square analysis of CD8-, PD1-, and PDL1-positive expression rate in 86 CRC tissues and 78 adjacent normal tissues.

To identify the molecular mechanism by which m6A modification promotes CRC progression, we also performed RNA sequencing (RNA-seq) of the CRC tissues and paired normal colorectal mucosa samples and combined the RNA-seq and MeRIP-seq results. RNA-seq revealed that 2358 genes were significantly upregulated (fold change >2 and *p* < 0.05), while 1758 genes were significantly downregulated (fold change <0.5 and *p* < 0.05), in CRC tissues compared to paired normal colorectal mucosa samples. MeRIP-seq revealed that the m6A peaks of 21 genes exhibited increased abundance (fold change >2 and *p* < 0.05) and 25 genes exhibited reduced abundance (fold change <0.5 and *p* < 0.05) in the CRC tissues compared to the normal mucosa samples. Intriguingly, 11 genes overlapped in the RNA-seq and MeRIP-seq results: DEGS2, ASB2, DPYSL3, SNX27, XYLB, MUC4, PTGDR, NBEA, DSTYK, OSBPL1A, LDB3 (Fig. 3a). As an example, the m6A abundance of DEGS2 was reduced by fourfold in CRC, and the *p* value was 0.034.

Next, we validated the mRNA level of these 11 candidate genes in CRC tissues and paired normal colorectal mucosa samples. We confirmed that DEGS2 was consistently upregulated in CRC tissues. The Cancer Genome Atlas (TCGA) data also confirmed that DEGS2 mRNA was significantly upregulated in CRC (Fig. 3b). We compared the mRNA levels of DEGS2 in 17 pairs of CRC and paired normal colorectal tissues by RT-qPCR. We found that the majority of CRCs exhibited significantly elevated expression of DEGS2 compared to that in adjacent normal tissues (Fig. 3c). Consistent with these findings, the protein levels of DEGS2 were significantly higher in human CRC tissues than in their paired normal gastric tissues by western blotting (WB) (Fig. 3d). Moreover, we investigated the relationship between DEGS2 expression and clinicopathological features in 32 CRC patients. DEGS2 expression was significantly interrelated with the American Joint Committee on Cancer (AJCC) stage and the CEA level (Table 1). The clinical OS/DFS survival analysis of DEGS2 in colorectal cancer is shown in Fig. S1.

**Table 1:** Clinical characteristics of 32 CRC patients depending on DEGS2 expression levels.

Feature	Degs2-high	Degs2-low	*n*	*p*
All cases	16	16	32	
Differentiation				0.32
Moderate-high	13	14	27	
Low	3	1	4	
AJCC stage				0.0092
Stage I & II	7	14	21	
Stage III	9	2	11	
Tumor size				0.67
≤5 cm	13	12	25	
>5 cm	3	4	7	
Microvascular infiltration				0.20
Absent	11	14	25	
Present	5	2	7	
Ki67 positive				0.55
≤60%	5	7	12	
>60%	10	9	19	
CEA				0.012
≤5 ng/mL	6	13	19	
>5 ng/mL	10	3	13	
Total cholesterol	4.66 ± 1.10	4.22 ± 0.71		0.20
Total bile acid	5.38 ± 4.80	4.54 ± 3.10		0.60
Free fatty acid	51.94 ± 22.66	45.69 ± 18.53		0.41

To investigate the expression of DEGS2 in CRC tissues, CRC tissue microarrays were used. Immunohistochemistry (IHC) analysis of the CRC tissue microarrays showed that DEGS2 was predominantly localized to the cytoplasm and significantly elevated in cancer tissues compared with normal tissues (Fig. 3e). We also explored the staining of CD8, PD1, and PDL1, which are checkpoint immunotherapy markers, by IHC. High DEGS2 expression was not correlated with CD8 T-cell infiltration or PD1 expression (*p* > 0.05) but was significantly negatively associated with PDL1 expression, which was mainly found on cancer cells and epithelial cells (*p* < 0.01, Fig. 3f). This indicated that the DEGS2 level was an independent risk factor for a poor prognosis in CRC patients who received checkpoint immunotherapy.

### The m6A-methylated coding sequence (CDS) regulates the level of DEGS2

**Fig. 4:**
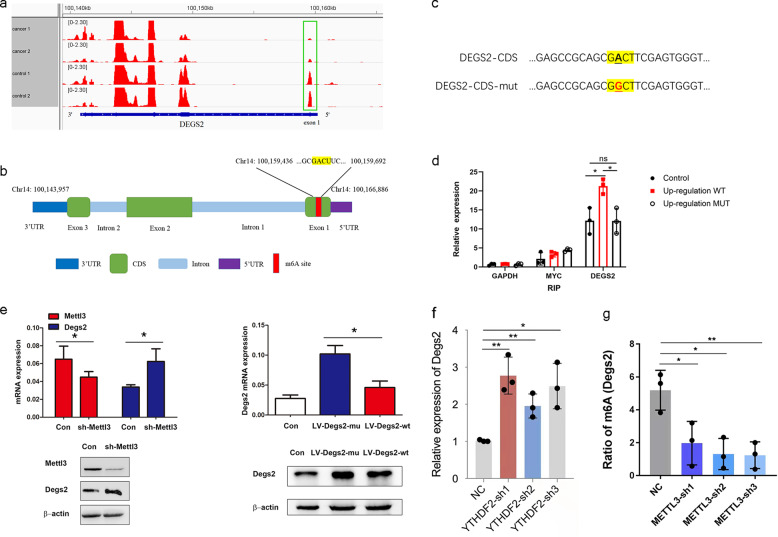
m6A methylation of the CDS regulates translation of DEGS2. **a** m6A peaks were enriched in the CDS and 3′ UTR of the DEGS2 according to the m6A-RIP-seq data. Squares indicate marked decreases in m6A peaks in CRC tissues compared to control tissues; **b** m6A-RIP assay confirmed the exact m6A location in the CDS region of DEGS2 mRNA. GAPDH mRNA was used as a nontarget control, and MYC mRNA was used as a positive control. ns, not significant; *p < 0.05. **c** Schematic representation of the position of the m6A motifs within DEGS2 mRNA. **d** Schematic representation of the mutation (GACT to GGCT) in the CDS to investigate the roles of m6A in DEGS2 expression. **e** sh-METTL3, LV-DEGS2-mut, and LV-DEGS2-WT were transfected into HCT116 cells for 24 h. Protein expression was measured by WB (bottom panel) and quantitatively analyzed (upper panel). **f** knockdown of YTHDF2 promoted the DEGS2 mRNA level. **g** knockdown of METTL3 reduced m6A methylation of DEGS2.

Our m6A-seq data confirmed that DEGS2 mRNA was modified by m6A and showed significantly reduced abundance of m6A in its CDS regions (exon 1) during CRC progression (Fig. 4a).

During CRC progression, the m6A levels of DEGS2 mRNA were significantly decreased, and the relative m6A enrichment in CRC was approximately 0.25-fold of that in control cells (*p* = 0.034). The data showed that m6A peaks located in CDS regions ranged from chr14:100,159,436 to 100,159, 692 (Fig. 4b). One GACU motif in the CDS was identified, as shown in Fig. 4b, which was consistent with the positions and numbers of peaks identified by m6A-seq.

By applying m6A RNA-immunoprecipitation (RIP) qPCR, we verified a 1.75-fold enrichment of DEGS2 mRNA with an m6A-specific antibody in cells transfected lentivirus expressing WT DEGS2 compared to control cells. We found no difference in DEGS2 mRNA enrichment with the m6A-specific antibody between the control cells and cells transfected with lentivirus expressing mutant DEGS2 (the potential m6A motif GACT mutated to GGCT, as showed in Fig. 4c), which verified this m6A motif in the CDS region of DEGS2 mRNA. We used GAPDH as a negative control and MYC, a classical m6A-modified gene, as a positive control. (Fig. 4d).

Mettl3 short hairpin RNA (sh-Mettl3)-mediated knockdown of METTL3 promoted the DEGS2 mRNA level, as determined by RT-qPCR, which was consistent with our m6A-RIP-seq data. In addition, WB analysis confirmed that METTL3 knockdown increased DEGS2 expression in HCT116 cells. Knocking down the writer Mettl3 increased DEGS2 mRNA and protein levels which, together with data in Fig. 4e showing that knockdown of Mettl3 reduced m6A methylation, suggests that Mettl3-mediated m6A modification is important for the stability/translation of DEGS2. We then investigated whether m6A methylation in the CDS can regulate the expression of DEGS2. We mutated the potential m6A motif GACT to GGCT (DEGS2-mut, Fig. 4e). HCT116 cells were transfected with DEGS2-CDS-WT or DEGS2-CDS-mut. Both qPCR and WB results showed DEGS2 expression in DEGS2-CDS-mut cells was higher than that in DEGS2-CDS-WT or control vector cells (Fig. 4e). Together, our data indicated that the DEGS2-CDS was the location for m6A-mediated expression regulation.

Several researchers have shown that YTHDF2 is a common reader that mediates the decay of m6A transcripts. We found that YTHDF2 short hairpin RNA (YTHDF2-sh)-mediated knockdown of YTHDF2 increased the DEGS2 mRNA level, which suggests that YTHDF2 can mediate degradation of m6A-modified DEGS2 mRNA (Fig. 4f). Our data also showed that Mettl3 short hairpin RNA (sh-Mettl3) mediated knockdown of METTL3 reduced DEGS2 m6A methylation and increased DEGS3 mRNA levels (Fig. 4e, 4). Taken together, we propose that both METTL3 and YTHDF2 are involved in regulation of m6A-modified DEGS2.

### DEGS2 promotes CRC proliferation and migration

**Fig. 5:**
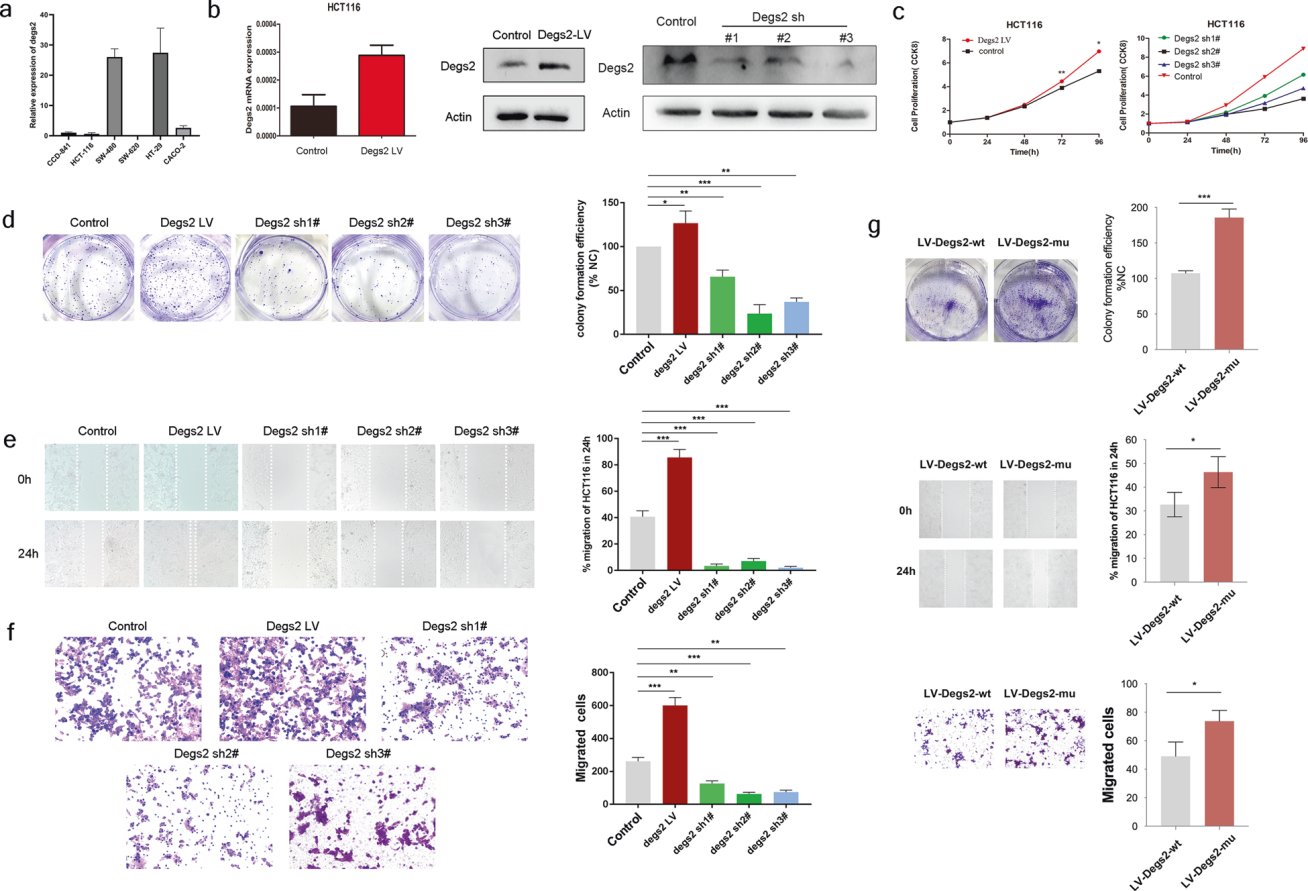
DEGS2 promotes CRC proliferation and migration. **a** DEGS2 mRNA levels in different CRC cell lines were verified by RT-qPCR. CCD-841 was selected as the control. **b** The DEGS2 overexpression and knockdown efficiency was verified at the mRNA (left panel) and protein levels (right panel) in HCT116 cells by qRT-PCR and WB assays, respectively. **c** CCK-8 assay of DEGS2-overexpressing and DEGS2 knockdown HCT116 cells. **d** Knockdown of DEGS2 impaired the colony formation ability, and overexpression of DEGS2 enhanced colony formation in HCT116 cells (left panel); quantification of the colony formation assay results (right panel). **e** The wound healing rate of cells transfected with wild-type (control) or DEGS2-overexpressing lentivirus cells, or DEGS2 knockdown shRNA was recorded (left) and quantitatively analyzed (right). **f** Representative images (left panel) and quantification (right panel) of the cell migration and invasion assay results for DEGS2-overexpressing and DEGS2-knockdown HCT116 cells transfected with DEGS2 shRNAs, lentivirus or their corresponding controls. Cells were allowed to invade for 24 h. **g** The colony formation, wound healing, and cell migration and invasion abilities of DEGS2-WT and DEGS2-mutant (GACT to GGCT in CDS motif) HCT116 were recorded (left) and quantitatively analyzed (right).

To examine the function of DEGS2 in CRC, we established stable DEGS2-overexpressing CRC HCT116 cells with DEGS2 overexpression lentivirus and constructed a DEGS2 knockdown cell line with shRNA and confirmed the efficiency of these methods by qPCR and WB (Fig. 5a).

The Cell Counting Kit-8 (CCK-8) assay showed that DEGS2 overexpression promoted cell proliferation while DEGS2 knockdown suppressed cell proliferation after 96 h of observation (Fig. 5b).

The upregulation of DEGS2 significantly promoted the colony formation ability of CRC cells, while shDEGS2-mediated knockdown of DEGS2 suppressed colony formation (Fig. 5c).

We evaluated the migration characteristics of DEGS2-overexpressing or DEGS2-knockdown cells. The results showed that both the wound healing (Fig. 5d) and in vitro invasion abilities (Fig. 5e) of DEGS2-overexpressing HCT116 cells were promoted compared with those of the control cells. Similarly, shDEGS2-mediated knockdown of DEGS2 suppressed the in vitro invasion of HCT116 cells. These data suggest that DEGS2 may act as an oncogene that promotes CRC proliferation and metastasis dependent on its biological activity.

To test whether mutation of the potential m6A motif (GACT to GGCT, DEGS2-mut) would affect the colony formation, wound healing, cell migration, and invasion abilities, we transfected cells with lentivirus encoding DEGS2-WT and DEGS2-mut. Mutation of the motif in the CDS region led to increases in the colony formation, wound healing, cell migration, and invasion abilities, suggesting that the m6A motif in the CDS region of DEGS2 mRNA is the location for expression regulation.

### Lipidomic alternations are closely related to the carcinogenesis of CRC due to DEGS2 dysregulation

DEGS2 is a key enzyme of Cer metabolism. To investigate the mechanism by which DEGS2 promotes CRC proliferation and migration, we employed lipidome analysis to clarify if lipid metabolism especially Cer metabolism is involved in this process.

Lipid metabolism is involved in energy transport, intercellular information communication and network regulation, and other necessary events during growth and development. Moreover, previous studies have demonstrated dramatic alterations in the metabolome and lipidome in human plasma caused by various diseases, including cancer and viral infections.

**Fig. 6:**
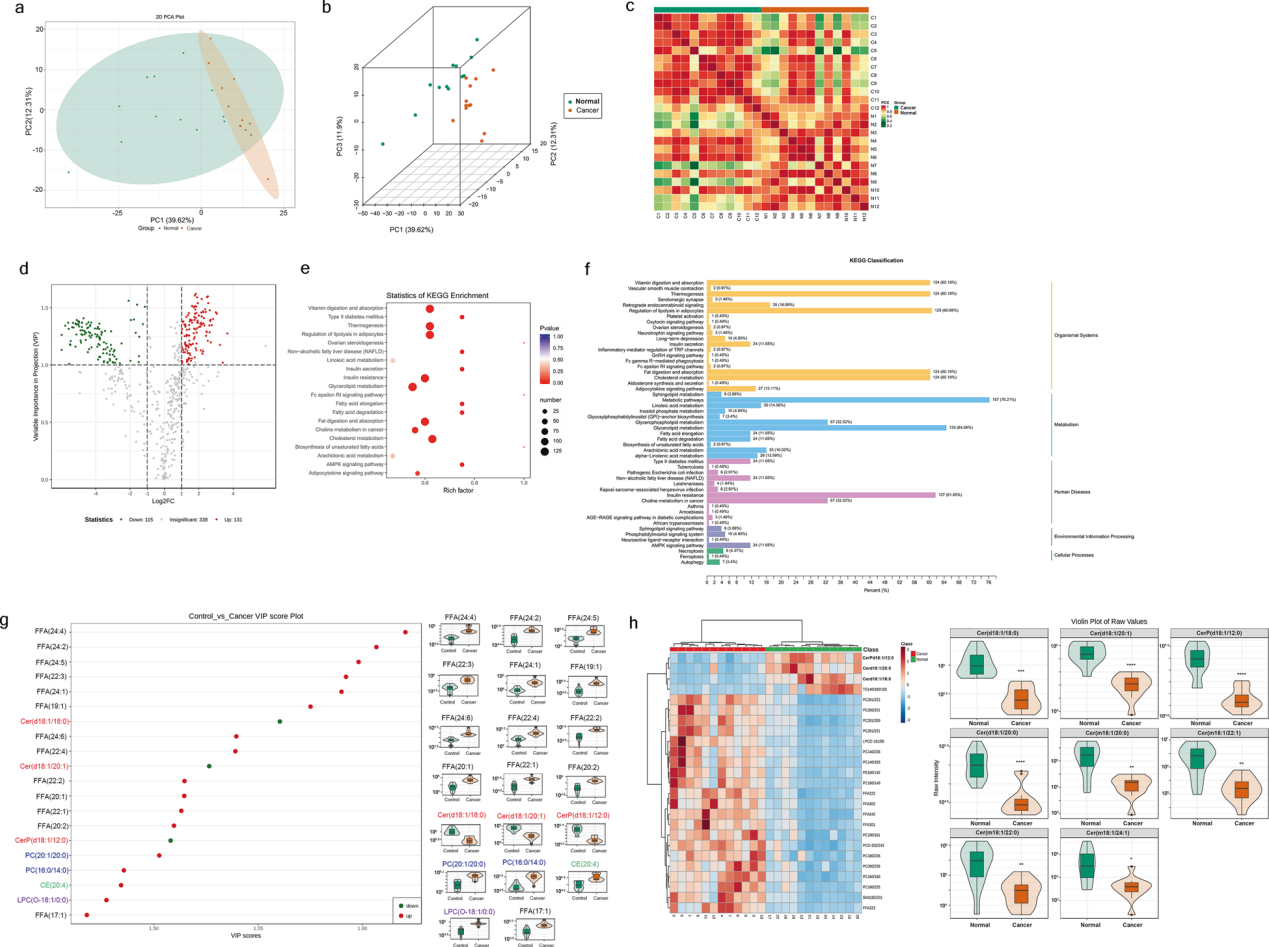
Lipidomic alternations were closely related to the carcinogenesis of CRC due to DEGS2 dysregulation. **a**, **b** Principal component analysis (PCA) used to show heterogeneity in lipid metabolism among samples from each group. **c** Pearson’s correlation coefficient calculation showed the correlation between the biological replicates. **d** Volcano plot showing the metabolites with twofold expression upregulation and VIPå 1 in CRC tissues compared with control tissues. **e**, **f** The cluster Profiler package identified the enriched KEGG and GO processes of 246 lipid metabolites that showed twofold expression upregulation and variable importance in projection scoreå 1 in CRC tissues compared with control tissues. **g** Top 20 metabolites in terms of VIP score, which were the most differentially expressed metabolites (left panel), and violin plots (right panel). **h** Heatmap of the top 20 most differentially expressed metabolites (left panel) and violin plots for 8 significantly altered ceramides.

Here, we performed targeted metabolomic and lipidomic profiling of tumor and tumor-adjacent samples collected from a cohort of CRC patients by ultraperformance liquid chromatography and tandem mass spectrometry (MS/MS). We investigated 21 major types of lipids, including eicosanoids, free fat acids (FFAs), lysolecithin (LPC, LPE, LPG, and LPI), sphingomyelin, Cer, cholesterol ester (CE), triglyceride, acylcarnitine, phosphatidic acid. Principal component analysis showed significant differences in the lipidome between CRC tissues and paired normal colorectal mucosa samples (Fig. 6a, b). We also found high heterogeneity in the lipidome of normal colorectal mucosa samples, while the CRC tissues showed a high level of uniformity (Fig. 6a). This was also confirmed by Pearson’s correlation coefficient analysis (Fig. 6c). A total of 584 lipid metabolites were included in our lipidome analysis.

Lipidome profiling identified 131 significantly upregulated lipid metabolites (fold change ≥ 2, variable importance in the projection [VIP] ≥ 1) and 115 downregulated lipid metabolites (fold change ≤ 0.5, VIP ≥ 1) (Fig. 6d). KEGG enrichment analysis of the 246 metabolites indicated that several metabolites were associated to vitamin digestion and absorption, thermogenesis, regulation of lipolysis in adipocytes, insulin resistance, glycerolipid metabolism, fat digestion and absorption, choline metabolism in cancer and cholesterol metabolism (Fig. 6e). KEGG classification analysis also showed that eight differentially expressed lipid metabolites were associated with sphingolipid metabolism and the sphingolipid signaling pathway (Fig. 6f).

The top 20 metabolites in terms of the VIP score (*p* value in the orthogonal projections to latent structures discriminant analysis [OPLS-DA] multivariable model involved in our lipidome analysis), which were the most differentially expressed metabolites, included 13 FFAs, 3 Cers, 2 PCs, 1 LPS, and 1 CE (Fig. 6g). The fold changes in the expression of Cer(d18:1/18:0), Cer(d18:1/20:1), and CerP(d18:1/12:0) were 0.24, 0.33, and 0.46, respectively, and the VIP scores were 1.56, 1.63, and 1.51, respectively. DEGS1 and DEGS2 share common substrate [15], dhCer. We speculate that enhanced DEGS2 could consume increased amount of dhCer, leading to reduction of Cer production by CRC cells. All three Cers were downregulated significantly, which were consistent with the hypothesis of decreased-m6A-induced DEGS2 upregulation in CRC.

A total of 33 Cers were included in the lipid metabolomics analysis, of which 8, including the three Cers above, showed statistically significant changes. The fold changes of the other Cers—Cer(d18:1/20:0), Cer(m18:1/20:0), Cer(m18:1/22:0), Cer(m18:1/22:1), Cer(m18:1/24:1) were 0.42, 0.07, 0.09, 0.07, and 0.07, and the VIP score was 1.36, 1.33, 1.20, 1.36, and 1.17, respectively. All 8 Cers were downregulated significantly as a result of DEGS2 upregulation in CRC (Fig. 6h).

### M6A methylation of DEGS2 regulates CRC proliferation and metastasis in vivo

**Fig. 7:**
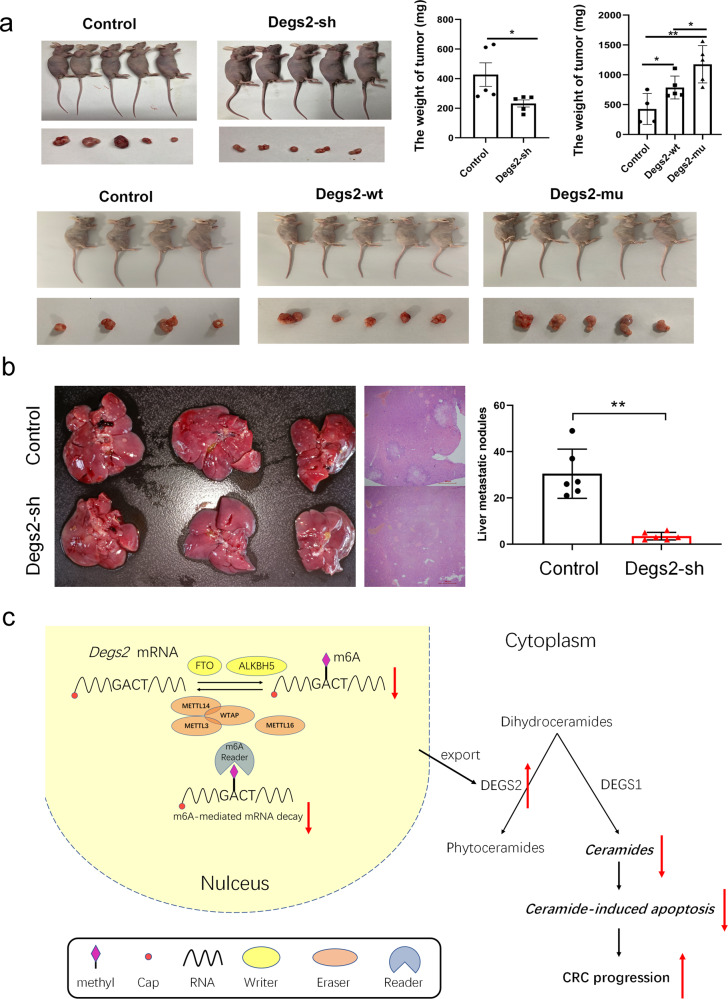
m6A methylation of DEGS2 regulates CRC proliferation in vivo. **a** Knockdown of DEGS2 effectively impaired the subcutaneous growth of CRC tumors in nude mice (*n* = 5), and DEGS2-mut promoted the subcutaneous growth of CRC tumors compared to DEGS2-WT. **b** The liver metastasis of HCT116 cells with knockdown of DEGS2 was significantly reduced compared with the negative control group. **c** Graphic illustration of the mechanism by which DEGS2 m6A methylation modulates tumor ceramide metabolism and promotes CRC tumor growth.

To confirm the role of DEGS2 in vivo, tumor xenograft models were constructed by subcutaneously injecting CRC cells with either stable knockdown of DEGS2(shDEGS2) into nude mice. We found that DEGS2 knockdown repressed tumorigenesis, as indicated by prominently lower tumor weights in the knockdown group than in the negative control group (Fig. 7a). Furthermore, to test whether the mutated DEGS2 m6A motif influences CRC tumor growth in vivo, we injected HCT116 cells with DEGS2-WT or DEGS2-mut (with mutation of the potential m6A motif GACT to GGCT). Tumor xenograft studies showed that DEGS2-WT promoted tumorigenesis, while DEGS2-mut induced an even stronger tumor proliferation ability than DEGS2-WT and the negative control.

To further clarify the effect of DEGS2 on the metastasis of CRC cells in vivo, we inoculated HCT116 cells with stable low expression of DEGS2 into nude mice by tail vein injection. We extracted liver tissues from nude mice 60 days after tail vein injection of the cells and detected the number of metastatic nodules in liver by HE staining. The liver metastasis of HCT116 cells with knockdown of DEGS2 was significantly reduced compared with the negative control group (Fig. 7b).

Taken together, these findings suggest that reduced m6A methylation promotes DEGS2 expression and leads to dysregulation of lipid metabolites, especially Cers, contributing to CRC proliferation and migration. The schematic diagram is showed in Fig. 7c.

## Discussion

Over 100 types of chemical modifications are present in human RNA [4, 16]. Among the RNA modifications, m6A is the most prevalent in mRNA [17]. A series of recent studies indicate that m6A modification is involved in various human diseases, including type II diabetes, cancer progression, viral infection and heart failure [18–22]. The m6A modification is dynamically regulated via m6A methyltransferase, demethylases and readers, which regulate RNA biological functions [23]. In the present study, we discovered that m6A mRNA methylation of DEGS2 regulates the Cer metabolism to control cell proliferation in CRC. We demonstrated that the m6A level is significantly increased due to downregulation of the m6A demethylases FTO and ALKBH5 and upregulation of the m6A reader protein IGF2BP3 in CRC. The combination of m6A-seq and RNA-seq revealed that 11 genes were targets of m6A dysregulation in CRC. In particular, DEGS2, a key enzyme of Cer metabolism, was m6A methylated in its CDS regions. Gain and loss of function and DEGS2 m6A motif mutation studies revealed that m6A in CDS of DEGS2 mRNA triggered its degeneration via interaction with methyltransferases. Knockdown of DEGS2 suppressed the proliferation and metastasis of CRC in vitro and in vivo, and conversely, its overexpression dramatically promoted the proliferation and metastasis of CRC in vitro and in vivo. Consistently, clinical data showed that tumor tissues with high DEGS2 levels had more advanced AJCC stage and higher serum CEA levels than those with low DEGS2 levels, which suggested a poor prognosis for CRC patients. Mechanistically, high DEGS2 expression suppresses Cer synthesis in cancer cells, which mediates the progression of CRC.

Using RNA-seq and MeRIP-seq, we found promising results indicating that DEGS2 was the key downstream target exhibiting m6A dysregulation in CRC. DEGS2, a DEGS1 homolog, is a key enzyme with C-4-hydroxylase activities that are responsible for the biosynthesis of phytosphingolipids. The tissue distribution profiles of the two enzymes are considerably different. DEGS1 is ubiquitously distributed, whereas DEGS2 is preferentially expressed in the intestine, skin, and kidney [24], where the production of phytoceramides is essential. DEGS1 and DEGS2 share a common substrate, dhCer, and elevated DEGS2 expression induced by decreased-m6A methylation leads to augmented production of phytoceramides and suppression of Cer production. Cer metabolism has been reported to be involved in numerous cancer-related processes, including cancer growth, apoptosis, angiogenesis and metastasis. However, knowledge on how DEGS2 regulates carcinogenesis remains elusive.

It was reported that the application of Cer analogs and ceramidase inhibitors induced rapid cell death in CRC through activation of various proapoptotic molecules, such as caspases and release of cytochrome c [25]. Ceramidase inhibition increases the Cer content of tumor cells, resulting in maximum activation of the apoptotic cascade. Moreover, treatment of nude mice with B13, the most potent ceramidase inhibitor, completely prevented liver metastasis of tumor using two different aggressive human colon cancer cell lines. We speculate that Cers act on CRC through apoptosis in this study.

Overall our data suggest that DEGS2 is an oncogene whose upregulation due to altered m6A methylation maybe important in the process of colon carcinogenesis through Cer signaling. Interestingly, among the genes that show altered m6A methylation in CRC, we also identified some that could serve as tumor suppressors. As an example, our meRIP showed that the m6A methylation of DPYSL3 was elevated and mRNA level was decreased in CRC. This leads us to wonder whether DPYSL3 is potential tumor suppressor and m6A methylation may promote tumor progression by reducing their mRNA levels. DPYSL3, which is a cell-adhesion protein, has been reported to be involved in the metastasis of tumors. In prostate cancer, overexpression of DPYSL3 decreased the cellular invasion and inhibited tumor metastasis [26]. Another study reported that DPYSL3 could reduce the ability of motility, migration, and invasion of lung cancer cells. DPYSL3 knockdown promoted TGFβ-induced EMT in lung cancer cells. Furthermore, the occurrence of metastasis was inversely associated with the expression level of DPYSL3 in lung cancer patients [27]. Consistently, our results showed DPYSL3 is a potential m6A-dependent tumor suppressor in CRC.

m6A modification affects RNA by not only reducing stability and translation, but also increasing stability and translation and affecting splicing. Recently, many studies choose one or two of m6A regulators to explore its aberrant expression and underlying mechanism in cancer. In HCC, Ma et al. have found that METTL14 positively manipulates primary microRNA126 splicing via an m6A-dependent manner, which impairs the metastatic potential of HCC. METTL14 affects the splicing stage of RNA and promotes the subsequent processing to mature miRNA [28]. In breast cancer, hypoxia facilitates expression of ALKBH5, resulting in reduction of NANOG mRNA m6A level and enhancement of mRNA stabilization. Elevated NANOG promotes breast cancer stem cells enrichment [29]. METTL3 stimulates AXL mRNA translation and epithelial–mesenchymal transition, thereby promoting growth and invasion of ovarian tumors [30, 31].

Recently, several studies started to uncover the role of m6A modification in CRC progression. A higher METTL3 level can promote colon cancer cell tumorgenicity by reducing suppressor of cytokine signaling 2 (SOCS2) expression in cancer cell lines [32]. Moreover, two independent groups claimed that m6A modification regulates glucose metabolism by stabilizing glucose transporter 1 (GLUT1) in CRCs [33, 34].

CRC is a major cause of cancer-related death worldwide. In developed countries, early detection through screening has improved the 5-year survival of patients with CRC, but ~25% of patients still present with stage 4 disease, and an additional 25–50% present with early-stage disease but eventually develop metastatic disease [35–38]. The prognosis of patients with metastatic CRC (mCRC) remains poor, with a median 5-year survival of only 12.5% in the USA [36]. Thus, the development of more effective treatments for patients with this disease is an urgent unmet need. In the past decade, immunotherapy has elicited tremendous excitement owing to its success in achieving long-term durable responses in previously difficult-to-treat solid tumors, such as melanoma and lung cancer. High tumor mutation burden has emerged as a marker of responsiveness to immunotherapy in several tumor types [39, 40]. In CRC, immune checkpoint therapy received regulatory approval in 2017 for the treatment of heavily mutated tumors that are mismatch repair deficient (dMMR) or have high levels of microsatellite instability (MSI-H) (termed dMMR–MSI-H tumors). By contrast, current immune checkpoint inhibitors are ineffective in tumors that are mismatch repair proficient (pMMR) and microsatellite stable or have low levels of microsatellite instability (MSI-L) (termed pMMR–MSI-L tumors). In these tumors, low tumor mutation burden and the lack of immune cell infiltration have been posited as mechanisms of immune resistance [41, 42]. We still lack biomarkers to predict immunotherapy effectiveness for CRC. Our results showed that DEGS2 expression is negatively correlated with PDL1 expression, indicating that DEGS2 is a promising biomarker for predicting the responsiveness to immunotherapy.

In summary, our findings reveal an oncogenic role of DEGS2 in CRC development. Mechanistically, m6A-dependent DEGS2 alteration promotes CRC tumorigenesis and metastasis by suppressing Cer metabolism. Moreover, DEGS2 expression is significantly increased in CRC tissues compared to normal tissues and is correlated with responsiveness to immunotherapy and a poor prognosis in patients with CRC. Therefore, DEGS2 might be a potential prognostic predictor and therapeutic target for CRC.

## Materials and methods

The details are described in Supplementary materials and methods. The online version of this article contains supplementary material and methods, which is available to authorized users.

## Supplementary information

supplementary materials and methods

Supplementary Figure 1

table S1

## Data Availability

All data generated or analyzed during the current study are included in this published article (and its supplementary information files) or available on published datasets (TCGA or GEO).
