# Supplementary material for: M6A methylation of DEGS2, a key ceramide-synthesizing enzyme, is involved in colorectal cancer progression through ceramide synthesis
Source: Oncogene. 2021 Aug 6;40(40):5913–24. doi: 10.1038/s41388-021-01987-z (PMC8497269; doi:10.1038/s41388-021-01987-z)
Supplement: Supplementary file 1 — supplementary materials and methods [file 41388_2021_1987_MOESM1_ESM.docx]

**Materials and Methods**

**Patients and specimens**

Samples of tumor tissues and adjacent tissues were gathered from 35 CRC patients who had received curative surgery from 2018 to 2019 at Qilu Hospital of Shandong University. All samples were clinically and pathologically verified. Frozen tissues were subjected to mRNA or protein extraction for reverse transcription quantitative real-time PCR (RT-qPCR) or western blotting analysis. Another cohort was based on CRC tissue microarrays (HCol-Ade180Sur-09), purchased from Shanghai Outdo Biotech, which contain 66 cases of gastric adenocarcinoma with paired paraneoplastic tissues, with one point for each tissue, and 8 cases of unpaired cancer tissues. This study was approved by Institutional Ethics Committee in Qilu hospital of Shandong University.

**Cell culture**

Human colorectal cancer cell lines SW620, HCT116, HT-29, SW480, Caco-2 and normal colon cell line CCD841 were purchased from Institute of Cell Research, Shanghai Cell Bank, Chinese Academy of Sciences. All cells were routinely cultured with Minimum Essential Media (MEM, Gibco) supplemented with 10% fetal bovine serum (FBS, Gibco) and incubated in 5% CO2, 37 °C incubator (Thermo Scientific, USA) with a humidified atmosphere.

**RNA extraction and RT-qPCR**

Total RNA was isolated using ESscience RNA-Quick Purification Kit (YiShan Biotech, Shanghai, China). Complementary DNA was generated from 1 μg of total RNA from tissues using cDNA synthesis with HiScript II Q RT SuperMix for qPCR (Vazyme Biotech, Nanjing, China). Expression of RNA was measured by SYBR Green (Vazyme Biotech, Nanjing, China) and normalized to Gapdh. All primers were derived from Tsingke Biological Technology (Beijng, China) and summarized in Additional file: Table S1.

**Western blotting**

Tissues or cells were washed in 1 mL of ice-cold PBS and pelleted at 400g. Pelleted were resuspended in 1 mL of pre-cooled RIPA buffer (Beyotime, Shanghai, China) containing protease and phosphatase inhibitors (Selleck, USA) and homogenized in a homogenizer. After clearing by centrifugation at 12,000g for 30 minutes, protein concentrations in the supernatant were quantified with Bicinchoninic Acid Protein Assay Kit (Thermo Scientific, USA). An equal amount of protein samples was separated by 10% SDS-PAGE (GenScript, Nanjing, China) using Bio-Rad system and then transferred to 0.45 μm PVDF membranes (Millipore, USA). The immunoblots were detected with an imaging system (Bio-Rad, USA) using enhanced chemiluminescence detection kit (Servicebio, Wuhan, China). Antibodies used include β-Tubullin (61157, proteintech), β-actin (TA336770, ZSGB-BIO), METTLE3 (A301-568A, Bethyl Laboratories) and DEGS2 (PA5-24082, invitrogen).

**Tissue chip and Immunohistochemistry**

The tissue chip (HColA180Su21) used in the present study comprised 74 colon cancer tissues and 66 corresponding adjacent colon tissues (1.5 cm away from the resection edge of the tumor; no residual tumor confirmed) and were purchased from Shanghai Outdo Biotech Co., Ltd. (Shanghai, China). The clinic pathological characteristics of the samples were available on the company’s website. The TMA cohort was utilized to construct the connection between DEGS2 and clinical characteristics of CRC patients. They were subjected to IHC staining using two-step method of Dako Envision™ Detection System (DakoCytomation, Glostrup, Denmark). The intensity and percentage of immunoreactive cells (immunoreactive scores) was computed by ImageJ system automatically. Finally, the immunoreactivity of the stained tissue samples was classified into low or high expression, respectively, according to median scores. The antibody used for IHC was as follows: DEGS2 (PA5-24082, invitrogen).

**Lentiviral transfection**

Lentiviral constructs repressing DEGS2, expressing DEGS2, repressing Metll3 and expressing mutant DEGS2(GACT mutant to GGCT in m6A motif of DEGS2 mRNA CDS region) were constructed and purchased from Genechem (Shanghai, China) and were used to establish HCT116 cell line and HCT116 cell line. Cells were infected with lentivirus for 24 h and selected by puromycin after 72h.

**Cell proliferation assay**

Cell proliferation ability was measured by Cell Counting Kit-8 (CCK-8, Beibo, Chinese). 5 × 10^3 cells were seeded into a 96-well plate per well with three duplications, followed by incubation for 4 h at 37 °C. Absorbance was detected at 450 nm daily for 5 consecutive days.

**Colony formation**

5× 104 treated cells were coated into 6-well plates with three repetitions. After 7-day incubation, these plates were washed with phosphate buffered saline (PBS) twice, fixed by methanol for 10 min and stained with 0.1% crystal violet solution within 10 min for further analysis.

**Migration and invasion assays**

Migration or invasion assays were performed using 24-well plates inserted by 8-μm pore size transwell filter insert (Corning, NY, USA) with or without pre-coated diluted Matrigel (1,10) (Becton Dickinson, San Jose, CA, US). 5 × 104 HCT116 cells with Serum-free medium were placed into the upper chamber, and medium containing 10% FBS was added into the bottom chamber subsequently. After incubation in 37 °C for 24 h (migration and invasion), cells on the underside of membrane were immobilized and stained with 0.1% crystal violet (Sangon Biotech, China). Then penetrated cells were counted in five random fields under the microscope.

**Cell migration**

The cell migration ability was accessed by wound-healing assay. Linear wound was generated with a 200 ul pipette tip until cell confluence. Wound closure was examined and photographed after 24 h in multiple microscopic regions.

**Subcutaneous xenograft experiments**

All animal experiments were approved by the Ethics Committee for Laboratory Animals of the Qilu Hospital of Shandong University. Four-week old Balb/c male nude mice were purchased from Beijing Vital River Laboratory Animal Technology Co, Ltd (Beijing, China) and randomly divided into five groups. After interference and reconstruction, HCT116 cells (control, Degs2-sh, Degs2-wt, Degs2-mu) were resuspended in 100 μl PBS with 1x 107 cells and subcutaneously injected to the right flank of the mice. To characterize tumors weight by balance scale and volume with the following formula: 1/2*(length×width2), mice were sacrificed at the end of feeding (one month). And extreme values (maximum and minimum) were eliminated.

**Liver metastasis model experiments**

To assess the efect of DEGS2 on cell metastasis in vivo, nude mice were subjected to a tail vein injection of 5× 10^6^ HCT116 cells (n=6 each group). Mice were euthanized using an intraperitoneal injection of sodium pentobarbital (150 mg/kg). After confrming the death of the mice, liver tissues were extracted from the mice for subsequent experiments. Collected liver tissues were fixed in 4% paraformaldehyde, embedded, and sectioned, followed by hematoxylin–eosin (HE) staining to count the number of metastatic nodules.

**RNA sequencing**

Total RNA was extracted and RNA purity was checked using the NanoPhotometer® spectrophotometer (IMPLEN, CA, USA). Then the library construction and RNA-sequencing (RNA-seq) were performed at Novogene Co.Ltd (Beijing, China) followed by the computational analysis they provided. The criteria for differential genes was set up with P value < 0.05 and fold change > 2 or < 0.5.

**m6A dot blot assay**

Total RNA or poly(A) + mRNA was isolated as described above. Determine the concentration of purified mRNA with NanoDrop and make a serial dilution of mRNA to 200 ng/μl, 100 ng/μl and 50 ng/μl using RNase-free water. Denature the serially diluted mRNA at 72 °C to disrupt secondary structures in a heat block for 5 minutes, followed by chilling on ice immediately to prevent the re-formation of secondary structures of mRNA. 2 μl of mRNA were loaded to an Amersham Hybond-N+ membrane (GE Healthcare, USA) optimized for nucleic acid transfer. The membrane was UV crosslinked in a Stratalinker 2400 UV Crosslinker twice using the Autocrosslink mode (1,200 microjoules, 25-50 sec) and washed with 10 ml of PBST in a clean washing tray for 5 min at room temperature with gentle shaking to wash off the unbound mRNA. After being blocked with 5% non-fat milk, the membrane was incubated with specific m6A antibody (Synaptic Systems,202003, 1:1000) overnight at 4 °C. Dot blots were hatched with HRP-conjugated anti-rabbit immunoglobulin G (IgG) for 1 h before visualized by an imaging system (Bio-Rad, USA), the other membrane was methylene blue stained as loading control.

**RNA immunoprecipitation (RIP)**

For m6A RIP, we used m6A antibody (Synaptic Systems, SYSY 202003 m6A antibody) to pull down m6A modified individual genes. Briefly, total RNA was extracted by EASYspin Plus kit (Aidlab) and dissolved in 40 µl RNase-free water, of which 2 µl was kept as RNA input, then the remaining RNA volume was adjusted to 1 ml buffer containing RNase inhibitor, ribonucleoside vanadyl complexes, m6A-specific antibody (Sigma-Aldrich) or rabbit IgG (Sigma-Aldrich), subsequently was rotated for 2 hr at 4 °C , then the prewashed beads A were added into the samples and reincubated for another 2 hr. Next, the elution buffer containing anti-m6A antibody (Synaptic Systems) was added to the mixture and incubated for 1 hr with continuous shaking at 4 °C. The methylated mRNAs were precipitated with one-tenth volumes of 3 mol sodium acetate in a 2.5 volume of 100% ethanol at -80 °C overnight. The m6A bound RNA was calculated by qPCR and the corresponding m6A enrichment was calculated by normalizing to the input.

**m6A sequencing (m6A-seq) and data analysis.**

The mRNA m6A was sequenced by MeRIP-seq at Novogene (Beijing, China). Briefly, a total of 300 μg RNAs were extracted from the CRC tissues and control. The integrity and concentration of extracted RNAs were detected using an Agilent 2100 bioanalyzer (Agilent) and simpliNano spectrophotometer (GE Healthcare), respectively. Fragmented mRNA (~100 nt) was incubated for 2 hr at 4℃ with anti-m6A polyclonal antibody (Synaptic Systems) in the immunoprecipitation experiment. Then, immunoprecipitated mRNAs or Input was used for library construction with NEBNext ultra RNA library prepare kit for Illumina (New England Biolabs). The library preparations were sequenced on an Illumina Novaseq or Hiseq platform with a paired-end read length of 150 bp according to the standard protocols. The sequencing was carried out with 3 independent biological replicates. Reference genome and gene model annotation files were downloaded from genome website directly. Index of the reference genome was built using BWA v0.7.12 and clean reads were aligned to the reference genome using BWA mem v 0.7.12. The m6A-enriched motifs of each group were identified by HOMER (version 4.9.1). Differential peak calling was performed using exomePeak R package (version 2.16.0) with parameters of P-value less than 0.05 and fold change more than 1. [[39](#_ENREF_39), [40](#_ENREF_40), [41](#_ENREF_41), [42](#_ENREF_42)]

**Lipidome analysis**

a. Tissue sample

Sample was thawed on ice. Take 50 mg of one sample and homogenize it with 1mL mixture (include methanol, MTBE and internal standard mixture) and steel ball. Take out the steel ball and whirl the mixture for 2 min. Add 500 uL of water and whirl the mixture for 1 min, and then centrifuge it with 12,000 rpm at 4 ℃ for 10 min. Extract 500 uL supernatant and concentrate it. Dissolve powder with 100 uL mobile phase B, then stored in -80 ℃. Finally take the dissolving solution into the sample bottle for LC-MS/MS analysis.

b. HPLC Conditions

The sample extracts were analyzed using an LC-ESI-MS/MS system (UPLC, Shim-pack UFLC SHIMADZU CBM A system, https://www.shimadzu.com/; MS, QTRAP® System, https://sciex.com/). The analytical conditions were as follows, UPLC: column, Thermo C30 (2.6μm, 2.1 mm*100 mm); solvent system, A: acetonitrile/water (60/40V,0.04% acetic acid, 5 mmol/L ammonium formate), B: acetonitrile / isopropanol (10/90 V, 0.04% acetic acid, 5 mmol/L ammonium formate); gradient program, A/B(80:20 V/V) at 0 min, 50:50 V/V at 3.0 min, 35:65 V/V at 5 min, 25:75 V/V at 9 min, 10:90 V/V at 15.5 min; flow rate, 0.35 ml/min; temperature, 45 ℃; injection volume: 2μl. The effluent was alternatively connected to an ESI-triple quadrupole-linear ion trap (QTRAP)-MS.

c. ESI-QTRAP-MS/MS

LIT and triple quadrupole (QQQ) scans were acquired on a triple quadrupole-linear ion trap mass spectrometer (QTRAP), QTRAP® LC-MS/MS System, equipped with an ESI Turbo Ion-Spray interface, operating in positive and negative ion mode and controlled by Analyst 1.6.3 software (Sciex). The ESI source operation parameters were as follows: ion source, turbo spray; source temperature 550 ℃; ion spray voltage (IS) 5500 V; ion source gas I (GSI), gas II (GSII), curtain gas (CUR) was set at 55, 60, and 25 psi, respectively; the collision gas (CAD) was medium. Instrument tuning and mass calibration were performed with 10 and 100 μmol/L polypropylene glycol solutions in QQQ and LIT modes, respectively. QQQ scans were acquired as MRM experiments with collision gas (nitrogen) set to 5 psi. DP and CE for individual MRM transitions was done with further DP and CE optimization. A specific set of MRM transitions were monitored for each period according to the metabolites eluted within this period.

**Statistical analysis**

All experiments were performed with biological replicates, with the exact sample size stated

in figure legends. All in vitro experiments were performed in triplicate, unless otherwise stated. Data were reported as mean ± SD from at least three independent experiments unless otherwise specified. Data were analyzed by two-tailed unpaired Student's t test between two groups and by one-way ANOVA followed by Bonferroni test for multiple comparison. Statistical analysis was carried out using SPSS 16.0 for Windows. All statistical tests were two sided. A p-value of < 0.05 was considered to be statistically significant. Data are presented as means ± SD from three independent experiments. *p < 0.05, **p < 0.01. NS, no significant.

**Figure legends**

**Supplementary Figure 1** The clinical OS/DFS survival analysis of DEGS2 in CRC.
