# Supplementary figures and images for: M6A methylation of DEGS2, a key ceramide-synthesizing enzyme, is involved in colorectal cancer progression through ceramide synthesis
Source: Oncogene. 2021 Aug 6;40(40):5913–24. doi: 10.1038/s41388-021-01987-z (PMC8497269; doi:10.1038/s41388-021-01987-z)

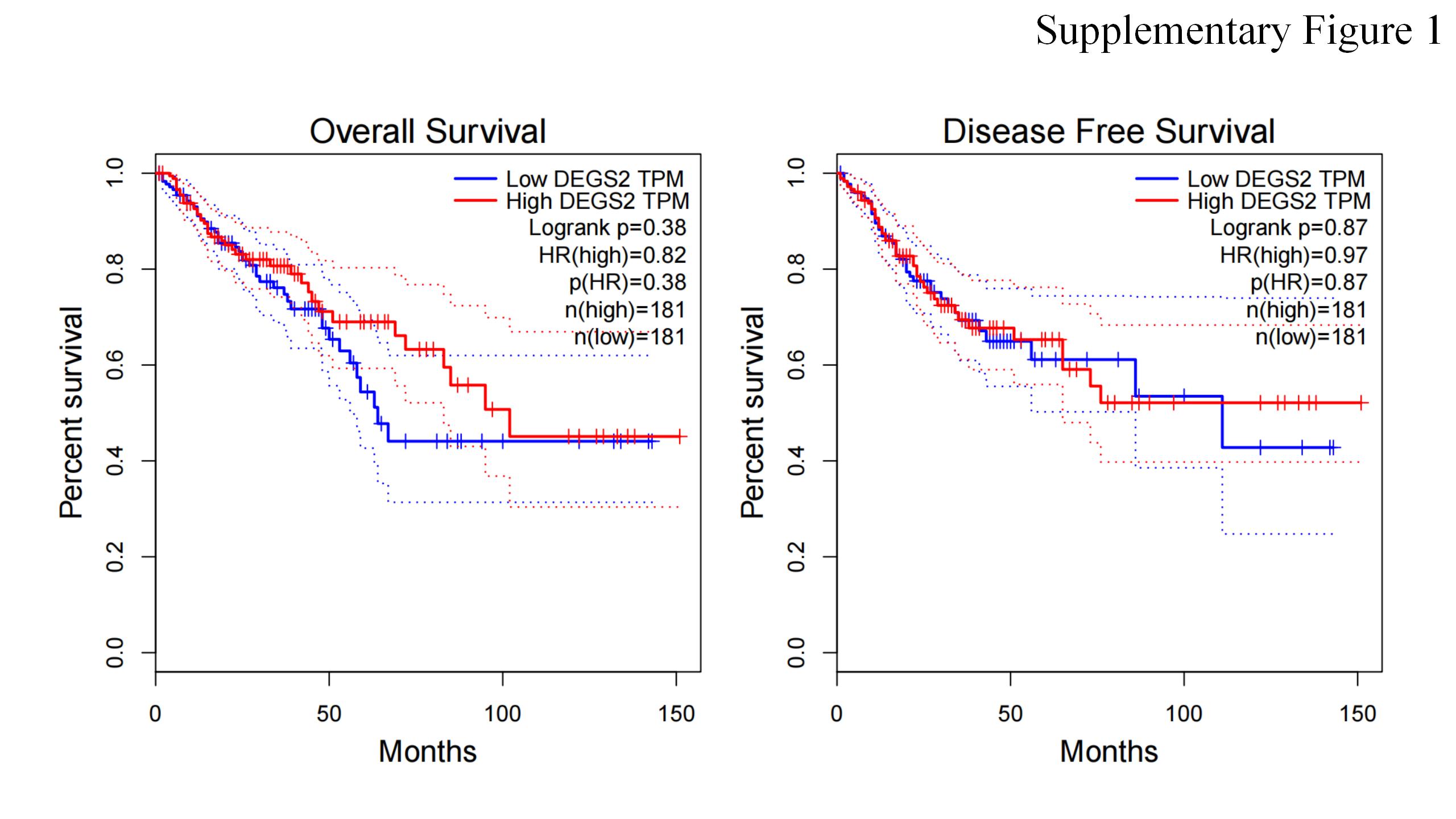

Supplement: Supplementary file 2 — Supplementary Figure 1 [file 41388_2021_1987_MOESM2_ESM.jpg]
